# Oxytetracycline reduces the diversity of tetracycline-resistance genes in the *Galleria mellonella* gut microbiome

**DOI:** 10.1186/s12866-018-1377-3

**Published:** 2018-12-29

**Authors:** Katarzyna Ignasiak, Anthony Maxwell

**Affiliations:** 1 Department of Biological Chemistry, John Innes Centre, Norwich Research Park, Norwich, UK; 2 Present address: School of Molecular Sciences, The University of Western Australia, 35 Stirling Highway, Crawley, Perth, 6009 Australia

**Keywords:** Antibiotic resistance, Tetracycline-resistance elements, Oxytetracycline, *Galleria mellonella*, Lepidoptera, Greater wax moth

## Abstract

**Background:**

Clinically-relevant multidrug resistance is sometimes present in bacteria not exposed to human-made antibiotics, in environments without extreme selective pressures, such as the insect gut. The use of antibiotics on naïve microbiomes often leads to decreased microbe diversity and increased antibiotic resistance.

**Results:**

Here we investigate the impact of antibiotics on the insect gut microbiome by identifying tetracycline-resistance genes in the gut bacteria of greater wax moth (*Galleria mellonella*) larvae, feeding on artificial food containing oxytetracycline. We determined that *G. mellonella* can be raised on artificial food for over five generations and that the insects tolerate low doses of antibiotics in their diets, but doses of oxytetracycline higher than sub-inhibitory lead to early larval mortality. In our experiments, greater wax moth larvae had a sparse microbiome, which is consistent with previous findings. Additionally, we determined that the microbiome of *G. mellonella* larvae not exposed to antibiotics carries a number of tetracycline-resistance genes and some of that diversity is lost upon exposure to strong selective pressure.

**Conclusions:**

We show that *G. mellonella* larvae can be raised on artificial food, including antibiotics, for several generations and that the microbiome can be sampled. We show that, in the absence of antibiotics, the insect gut microbiome can maintain a diverse pool of tetracycline-resistance genes. Selective pressure, from exposure to the antibiotic oxytetracycline, leads to microbiome changes and alteration in the tetracycline-resistance gene pool.

**Electronic supplementary material:**

The online version of this article (10.1186/s12866-018-1377-3) contains supplementary material, which is available to authorized users.

## Background

Antibacterial resistance is manifested by the ability of bacteria to grow and multiply in the presence of an antibiotic that would normally kill them or limit their growth. It can be caused by three different mechanisms: a target mutation (where the antibiotic target changes so that it is no longer sensitive to the compound), efflux pumps (which remove the antibiotic or toxin from the bacterial cell) or an antibiotic-modifying enzyme (where a separate protein capable of deactivating the antibiotic is present) [1]. Resistance by target mutation requires point mutations to arise spontaneously, and such alleles can be commonly found in natural environments with no selective pressure [2]. Surprisingly, efflux pumps and modifying enzymes also can be found in environments with no antibiotic exposure, such as permafrost samples [3], glaciers [4], or the insect gut [5].

Antibiotic use in agriculture has had a profound effect on bacterial populations associated with insects. Oxytetracycline is the most commonly used antibiotic in apiculture and the long-term exposure of bees to this antibiotic has led to abundance of tetracycline-resistance genes, conferring resistance to both tetracycline and oxytetracycline, in the bee gut microbiota [6]. Oxytetracycline is used in the control of European foulbrood, a disease of honeybees caused by *Melissococcus plutonius*. The antibiotic is applied mixed with sugar or water and reaches minimal inhibitory concentrations in the larval guts within 24 h post treatment, but declines rapidly to a level under MIC within two to five days [7, 8]. In the USA, bees have been exposed to prophylactic oxytetracycline use for over 50 years and high levels of tetracycline resistance can be detected in bee guts [6]. In contrast, tetracycline-resistance genes are rare and are only present at low frequencies in bees not exposed to antibiotics (from countries where antibiotics are not permitted in apiculture) and wild bumblebees. Together these studies suggest that the exposure of insect microbiota to antibiotics leads to an accumulation of antibiotic resistance.

To further study this phenomenon, we exposed greater wax moth (*Galleria mellonella*) larvae to oxytetracycline over five generations. *G. mellonella* is a host commonly used in immunity research, bacterial virulence and antibiotic efficacy studies (Additional file 1: Figure S1) [9]. The larvae are large and easy to dissect, with the fifth instar reaching up to 30 mm long. They are robust and can be readily utilised in antibiotic testing and acute toxicity trials [10]. In their natural environment greater wax moths are pests of beehives and feed on honeycomb or beehive debris [11]. However, these Lepidoptera can also be reared on bran- and wheat germ-based artificial food. The gut microbiota of *G. mellonella* has been identified as simple, containing mainly one bacterial species: *Enterococcus faecalis* (syn. *Streptococcus faecalis*) [12]. More recently culture-independent methods confirmed the presence of Enterococcus species in the larval guts, but identified the dominant strain as *Enterococcus munditii* [13]. The strain has no role in insect digestion [14], but together with the host provides colonisation resistance against invading pathogens [15]. However, it is important to appreciate that diet diversity has an effect on gut microbiome composition [16], as well as other parameters, such as expression of some stress-related genes and antimicrobial peptide genes [17].

In this study we exposed *G. mellonella* to oxytetracycline over five generations in order to test the hypothesis that compounds with antibacterial activity in the insect food select antibiotic resistance in the insect gut microbiome. The aim of this project was to investigate the correlation between the antimicrobial compounds in the insect food and the presence of antibiotic resistance elements in the insect gut.

## Results

### *G. mellonella* can be fed on artificial food supplemented with antibiotics

To assess if *G. mellonella* can be raised on artificial food over multiple generations, a colony was set up feeding on bran-based artificial food (Additional file 1: Figure S1). The larvae adapted readily, reached fifth instar, and pupated. Healthy adult moths emerged from the pupae and laid eggs, which were moved to fresh food for the larvae to hatch on. The entire life cycle lasted ~ 6 weeks. Moths were reared on artificial food over five generations, which allowed us to perform longitudinal studies.

To assess the viability of *G. mellonella* on artificial food containing antibiotics, the food was spiked with oxytetracycline at concentrations varying from sub-inhibitory to toxic. Antibiotic doses were selected to reflect a range of physiologically-relevant human doses. Oxytetracycline is recommended for human use at 15 mg/kg body weight/day. The lowest dose (1 mg/100 g artificial food) is representative of a sub-inhibitory antibiotic dose. The 10 mg/100 g diet oxytetracycline dose represents a high therapeutic dose. The two highest doses are above the dose recommended for therapeutic use and are high enough to have toxic effects on the larvae. Larvae feeding on lower concentrations of the antibiotic reached maturity, pupated, and the adults laid viable eggs. The first instar larvae were healthy and continued feeding on the artificial diet (Table 1). We consistently observed that the insects feeding on 1 mg/100 g food were larger than the larvae feeding on food with no antibiotic. The viability of insect reared on artificial food with a low dose of oxytetracycline allowed us to collect gut samples from insects exposed to antibiotics over five generations. It should be noted that as the larvae were feeding on food supplemented with antibiotics, the actual antibiotic dose is likely to vary between individuals. However, this emulates the natural situation where the larvae are potentially feeding in beehives treated with antibiotics.

**Table 1:** *G. mellonella* larvae collected from the feeding procedure

Oxytetracycline dose ⇓	Generation ⇒	1	2	3	4	5
0		+	+	+	+	+
1 mg/100 g		+	+	+	+	+
10 mg/100 g		+	+	–	–	+
100 mg/ 100 g		+	–	+	–	+
400 mg/ 100 g		+	–	+	–	+

At least 10 larvae were collected per feeding group. In some cases the larvae in an antibiotic-treated group died (−) and not only could no larvae be collected, but also no larvae reached maturity and laid eggs. In the interest of continuing the experiment, in such cases eggs laid by the adults from the control (no drug) group were moved to the food spiked with oxytetracycline. (+) indicates survival and that a sample could be collected

### Therapeutic doses of oxytetracycline lead to early larval mortality

When testing different doses of oxytetracycline in the insect food to determine a suitable dose for a longitudinal study, we observed that oxytetracycline doses equal to or higher than 10 mg/100 g food led to increased mortality (Table 1). The first and second generation of insects feeding on a diet containing a therapeutic dose of oxytetracycline (10 mg/100 g food) reached maturity and laid eggs. The third generation of larvae did not reach fifth instar stage, at which we sampled the insect guts. Similarly, the first generation of larvae feeding on the subtoxic (100 mg/100 g food) and toxic (400 mg/100 g food; Table 1 [10]) dose reached maturity, but the larvae that hatched in the second generation were not viable. The increased mortality of insects feeding on therapeutic and higher doses of oxytetracycline resulted in limited data available for assessing the impact of those doses on the insect gut microbiome.

### The microbiome of *G. mellonella* has low abundance and low diversity

The gut contents of *G. mellonella* fifth instar larvae were assessed by culture-dependent and culture-independent methods. A proportion of the gut contents, that has been previously shown to be sufficient to produce visible colonies (data unpublished), was plated on a selection of bacteriological media. Only two plated samples produced visible colonies: a sample from the initial stock insects had two oxytetracycline-sensitive *Enterococcus* species, and a sample from the fourth generation of larvae feeding on 1 mg/100 g food had an oxytetracycline-resistant strain of *Bacillus subtilis*. The culturing methods were optimised for typical *G. mellonella* microbiome and the low number of strains cultured from the insect guts indicates that the guts harboured a sparse microbiome. To further explore bacterial abundance and diversity we isolated total DNA from the insect guts. Detectable levels of genomic DNA were present in 57% of the guts at concentrations ranging from 10 to 230 ng/μL, suggesting that nearly half of the guts did not carry any bacteria, and the remaining guts often had low abundance of gut bacteria. Together the low number of bacterial strains isolated from the guts of *G. mellonella* and the low amounts of DNA recovered from the guts indicate that the microbiome was low in abundance and diversity, which is consistent with previous findings [13].

### Tetracycline-resistance genes were present in the guts of insects not exposed to antibiotics

To assess the level of tetracycline resistance in the insect guts, the genomic DNA isolated from *G. mellonella* larvae was surveyed for the presence of tetracycline-resistance genes (Additional file 2: Table S1). The tetracycline-resistance gene *tetM* was found in the guts of the initial stock larvae, which had not been exposed to antibiotics and which harboured oxytetracycline-sensitive *Enterococcus* species. Additionally, tetracycline-resistance genes *tetB, tetC, tetD, tetL*, and *tet30*, which encode efflux pump proteins, were found in the guts of larvae feeding on artificial food not containing oxytetracycline (Table 2). The presence of antibiotic-resistance genes in the insect guts not exposed to antibiotics shows that these genes can be present in the absence of selective pressure.

**Table 2:** An overview of tetracycline-resistance elements from the guts of *G. mellonella*

	Tetracycline-resistance gene isolated from *G. mellonella* guts				
Generation ⇒	1	2	3	4	5
Oxytetracycline dose ⇓					
0	*tetB, tetC*	tetB, tetC	tetB, tetC, tet30	tetB, tetD	tetb, tetD, tetL
1 mg/100 g	tetB	tetB, tetD	tetB, tetD	tetB, tetD	tetB, tetD
10 mg/100 g	tetB, tetD	tetB, tetD	–	–	tetB, tet30
100 mg/100 g	tetB	–	Degraded gDNA	–	No *tet* genes
400 mg/100 g	tetD	–	No *tet* genes	–	No *tet* genes

There were no samples available to assess for groups of larvae with high mortality (−). For the third generation of larvae feeding on 100 mg/100 g food we were able to collect the gut sample, but only degraded DNA was present, probably due to shearing

### Oxytetracycline in the insects’ diet leads to a reduction in the diversity of tetracycline-resistance genes

To assess the impact of long-term oxytetracycline exposure on the insect gut microbiota, we surveyed the tetracycline-resistance genes in the genomic DNA isolated from the larvae feeding on oxytetracycline. In the guts of *G. mellonella* larvae feeding on 1 mg/100 g food we identified *tetB* and *tetD* genes (Table 2). The same genes were found in the guts of first and second generation of larvae feeding on 10 mg oxytetracycline/100 g food. The insects did not reach maturity in the third and fourth generation, but in the guts of larvae in the generation corresponding to the fifth we identified *tetB* and *tet30* genes. In the guts of first generation insects feeding on 100 and 400 mg/100 g food we identified *tetB* and *tetD* genes, respectively. All of the detected genes: *tetB*, *tetD*, and *tet30* encode efflux pumps. Compared to the genes present in the guts of insects not exposed to antibiotics, there are fewer different tetracycline-resistance genes present in the guts of insects feeding on oxytetracycline, indicating that in the presence of a strong selective pressure some genes are more likely to be retained in the microbiome than others.

### Metagenomic analysis of the *G. mellonella* gut microbiota

To further explore the *G. mellonella* gut microbiome, the genomic DNA samples were sequenced. Out of 25 samples that have met the sequencing criteria two successfully went through amplification with the sequencing primers specific for bacteria: one from the initial set of larvae used to set up the colony, and another from the first generation of larvae feeding on 400 mg oxytetracycline/100 g food. Nearly 20,000 valid reads were recovered from each sample, with the antibiotic-free stock larvae reads assigned to 2673 operational taxonomic units and the reads from larvae fed 400 mg oxytetracycline/100 g food assigned to 4310 operational taxonomic units (Additional files 3 & 4: Figures S2 & S3). Both samples were analysed for alpha-diversity, which indicates the biodiversity of a single community, in our case each sample containing DNA from three larval guts. Even though a similar number of reads was recovered from both communities, the oxytetracycline-fed larvae guts yielded nearly twice as many different operational taxonomic units, suggesting the gut communities from antibiotic-fed larvae were more diverse than in the antibiotic-free larvae. This result supports the hypothesis that oxytetracycline disrupts the simple native gut microbiota, which provides colonisation resistance, and allows establishment of a more diverse microbial community.

Each operational taxonomic unit was identified using the EzTaxon database by finding the closest related strain. Nearly all reads from the antibiotic-free sample belonged to one phylum, the Firmicutes, and it was the only identifiable phylum of bacteria present in the sample (Additional file 4: Figure S3). The remaining reads were eukaryotic, plant DNA from the *G. mellonella* food, and unmatched sequences, which had less than 97% similarity to any sequence in the database. This finding is consistent with previous data that *G. mellonella* microbiome is monoxenic [11–13]. On the species level the gut microbiome from the oxytetracycline-free sample was dominated by *Enterococcus mundtii*, and the remaining strains were: strain *Enterococcus dispar*, EU465963_s, and Enterococcus_uc. All remaining strains were present below 1% abundance. These results confirm that Enterococcus species dominate the gut of *G. mellonella* when the insects are fed antibiotic-free diets.

The sequencing reads from the oxytetracycline-fed larvae guts were more diverse, with 95% bacterial reads and 3% archaeal reads with the remaining 2% reads belonged to Eukaryota and unmatched sequences (Additional file 4: Fig. S3). Within the bacterial reads, not only Firmicutes (81% total reads) were present, but also Proteobacteria (4%), Thermotogae (3%) and Bacteroides (1%) suggesting that the exposure to antibiotics disrupts the monoxenic microbiome typically associated with *G. mellonella* gut. The gut community was dominated by strain EU465963_s, with strain Enterococcus_uc and strain Enterococcus_uc_s also present. The remaining strains were present below 1% abundance, with *Enterococcus mundtii* detected at 0.03%, which is consistent with the antibiotic disruption of the typical microbiome.

## Discussion

### *G. mellonella* can be raised on artificial food, including artificial food containing antibiotics

In our experiments *G. mellonella* larvae were feeding on artificial food with and without antibiotics for five generations. These results are consistent with the assessment of different cost-cutting modifications to the artificial diet, which determined that the larvae are capable of surviving on both honey- and honey waste-based diet and on different proportions of maize to wheat flour [18].

Additionally, we observed that insects feeding on a sub-inhibitory dose of oxytetracycline were larger than larvae not exposed to antibiotics. This finding is consistent with previous experiments with penicillin, streptomycin, and griseofluvin in *G. mellonella* in which the wet weight of larvae feeding on low doses of antibiotics increased [19], as well as with the murine model for the antibiotic-mediated weight gain in farm animals [20]. The use of antibiotics as growth promoters for farm animals is a controversial topic and evidence suggests that this practice contributes to the rise in antimicrobial resistance in humans [21]. That we found *G. mellonella* larvae feeding on food containing antibiotics were larger than control larvae suggests that *G. mellonella* could be used as a model system to study the use of antibiotics as growth promoters.

### High doses of oxytetracycline cause higher mortality

There was high mortality in the groups of *G. mellonella* larvae feeding on artificial food with high concentrations of oxytetracycline. Mortality associated with antibiotics in the artificial food is often due to the loss of obligate bacterial symbionts. However, *G. mellonella* can be grown axenically, and the larvae are healthy and able to digest their diets as efficiently as non-axenic cultures [11]. It is more likely that the mortality in the groups of wax moth larvae exposed to high antibiotic concentrations was due to oxytetracycline toxicity. Oxytetracycline administered orally is toxic to rats at 4800 mg/kg body weight [10] and the highest oxytetracycline doses fed to *G. mellonella* larvae was close to this concentration, making it likely that the larvae died because of poisoning. The highest doses were selected as part of a wide range of concentrations, spreading from a sub-inhibitory concentration to a toxic dose, in an attempt to capture the impact of antibiotics on the insect gut microbiota at different physiological concentrations.

### *G. mellonella* larvae have sparse microbiome

Only three strains were cultured from two samples of *G. mellonella* guts, even though nearly 200 guts were sampled. The control sample collected before the experiment commenced contained two Enterococcus strains, which constitute typical wax moth microbiota [14]. The strains were oxytetracycline-sensitive. The presence of such isolates is consistent with previous studies, showing that *Galleria mellonella* is a monoxenic insect associated with Enterococcus species [12, 13, 16]. When assayed for antibiotic susceptibility, the strain is normally resistant to β-lactam antibiotics and susceptible to tetracyclines [15].

A tetracycline-resistant *B. subtilis* strain was identified from the guts of fourth generation larvae feeding on oxytetracycline at 1 mg/100 g food. This dose of oxytetracycline is approximately half of the recommended human dose. The presence of tetracycline-resistant *B. subtilis* indicates that sub-inhibitory antibiotic concentrations can select antibiotic-resistant phenotypes. It is possible that the sub-inhibitory dose was harming native microbiota and allowing other strains to enter the niche.

### Metagenomic analysis of the larval guts

The metagenomic analysis of *G. mellonella* gut contents adds an extra dimension to what was already known about the composition of its gut microbiota. Previously described as having extremely simple gut community composed of one member species [12, 14], antibiotic-free *G. mellonella* harbours a community dominated by a subset of closely-related Enterococcus species. The community is dominated by *Enterococcus mundtii*. *G. mellonella*-derived Enterococcus species have been previously described as antibiotic-producers preventing colonisation by other species [13, 22], and our results confirm low abundance of other bacteria consistent with this hypothesis. However, it is not possible to hypothesise further about other potential roles of Enterococcaceae in *G. mellonella*.

For both *G. mellonella* gut samples the phylum Firmicutes was dominated by the Enterococcaceae family. This finding supports previous culture-dependent studies that have found only Enterococcus species in *G. mellonella* gut [12]. In previous studies predominantly *Enterococcus faecalis* (syn, *Streptococcus faecalis*) was isolated, but it was later identified to be more likely a strain of *Enterococcus faecium* (syn. *Streptococcus faecium*) [23]. Previous culture-independent studies described a gut community composed of Enterococcus species, dominated by *Enterococcus mundtii* [13]. In our study we found a diverse set of *Enterococcus* species.

The bacterial diversity discovered in the guts of *Galleria mellonella* feeding on artificial food with and without oxytetracycline is higher than the diversity described before. It is possible that the insects sampled in the previous studies were not monoxenic. The guts might have contained a variety of closely-related Enterococcus strains that are difficult to distinguish, without modern molecular biology techniques and sufficient sampling depth. Even with sensitive PCR methods, if not enough reads are sequenced, the microbial community might appear as monoxenic.

### Tetracycline-resistance genes are present in the absence of antibiotics in the insect food

A variety of tetracycline-resistance genes was identified from antibiotic-free *G. mellonella* larvae. The initial stock of insects used to start the colony carried only the *tetM* gene, which was the only ribosome-protection protein. The gene product of *tetM* complexes with 70S ribosome and directly blocks the tetracycline-binding site [24]. It is hypothesised the *tetM* ribosome protection protein dislodges tetracycline from the tetracycline binding site to confer resistance [24]. Interestingly, the bacteria cultivated from the same guts were tetracycline-sensitive, even though the tetracycline-resistance gene *tetM* was detected in the sample.

The microbiomes from other antibiotic-free larvae did not carry genes coding for ribosome protection proteins, but only genes coding for efflux pumps: *tetB, tetC, tetD, tetL*, and *tet30*. All these genes belong to group 1 tetracycline efflux proteins [25], which is unsurprising as these genes are mostly found in Gram-negative strains, such as the Enterococcus strains dominating the *G. mellonella* gut community. These results suggest that in the absence of antibiotics the antibiotic-resistance gene pool is diverse and dynamic.

### Fewer tetracycline-resistance genes are present in the guts on insects exposed to antibiotics

Three tetracycline-resistance genes were detected in the guts of insects feeding on oxytetracycline: *tetB, tetD*, and *tet30*. Both *tetB* and *tetD* were present in six groups, *tetB* alone was present in two guts, one group contained *tetB* and *tet30* combination and another group contained *tetD* gene alone. The most commonly found gene was *tetB*, which is unsurprising as *tetB* has wider resistance spectrum and wider host range than other group 1 tetracycline efflux proteins [25]. *TetB* is the most commonly carried tetracycline efflux pump among Gram-negative bacteria [26]. The other genes, *tetD* and *tet30*, are typical representatives of group 1 tetracycline resistance efflux pumps. A pool of antibiotic resistance genes was present as a baseline in insects not exposed to antibiotics, and adding the selective pressure selected for the most common one with the widest resistance range.

## Conclusions

Our aim was to test whether the microbiomes of insects feeding on compounds with antibiotic activity acquire antibiotic resistance. We identified oxytetracycline-sensitive bacterial strains in the guts of larvae unexposed to oxytetracycline and an oxytetracycline-resistant strain from larvae feeding on a sub-inhibitory dose of the antibiotic. A survey of tetracycline resistance genes present in the larval guts identified a number of different efflux pumps in the guts of insects feeding on artificial food without antibiotics and a smaller sub-set of these efflux pumps in the guts of insects feeding on artificial food containing oxytetracycline.

These results show that a baseline diversity of tetracycline-resistance genes exists in the larval guts not exposed to antibiotics. From this pool, genes are rapidly selected upon exposure to antibiotics, at a range of doses: sub-inhibitory concentrations, therapeutic doses, and toxic doses.

We have demonstrated that the antibiotic resistance genes are not necessarily acquired by the insect gut bacteria in response to a selective pressure. Instead a few resistance genes are selected from a diverse and dynamic pool of genes already present in the microbiome. Such a dynamic resistome in the guts of agriculture-associated insects can contribute to the dissemination of resistance genes in the environment.

## Methods

### Insect husbandry

A colony of *G. mellonella* was obtained from the John Innes Centre (JIC) Insectary. The colony was kept in the dark at 30 °C. The larvae were kept in large Petri dishes (140 mm, Sterilin) filled with artificial food. The artificial food was composed of 300 mL honey (Sainsbury’s Honey, Clear), 400 mL glycerol (G5516, Sigma Chemicals), 200 g milk powder (Dried Skimmed Milk Powder, Marvel), 200 g wholemeal flour (Strong Stoneground 100% Wholemeal Flour, Sainsbury’s), 100 g yeast powder (103,753, Merck), 100 g wheat germ (Neal’s Yard Wholefoods Natural Wheatgerm) and 400 g bran (Neal’s Yard Wholefoods Natural Wheat Bran). First, the dry and wet ingredients were mixed separately, and then the mixtures were combined. The diet was mixed with beeswax pellets at a 2:1 ratio. Unused food was stored at 4 °C. Oxytetracycline was added to the glycerol batches at 1 mg/100 g diet dry weight, 10 mg/100 g diet dry weight, 100 mg/100 g diet dry weight, and 400 mg/100 g diet dry weight. Estimating that 20 larvae fed on 25 g artificial food for a week, the lowest dose (1 mg/100 g food) equals approximately 8 mg/kg body weight oxytetracycline. The food was prepared fresh and replaced at least once per week, unless not enough was left for the larvae to feed on, in which case more food was added to the containers.

The *G. mellonella* colony was divided into five groups, each group feeding on a different diet. For every generation ten fifth instar larvae from each group were collected for gut dissection. The larvae were frozen in liquid nitrogen and stored at − 80 °C. The remaining larvae were moved to larger clean containers to pupate. The moths laid eggs along the edges of the lining of the containers. Eggs were collected and placed on fresh food with or without antibiotics. This procedure was followed for five generations. We started with 20–30 larvae per feeding group. Either they lived, in which case we would keep the group roughly that size (up to 50–60), or they died. We collected samples from all generations (where possible) at fifth larval instar (immediately before pupation).

Larval mortality was high in the groups feeding on 10 mg OTC/100 g diet or more, leading to cases when no individuals feeding on those oxytetracycline concentrations were left. In such cases, a new colony was established from antibiotic-free larvae on the appropriate antibiotic diet.

### Insect dissection

All steps of the dissection procedure were carried out on ice and in a biological safety cabinet. A sterile Petri dish was used as a dissecting surface. One larva was dissected at a time, to prevent defrosting of the samples. The head of a larva was cut off, while the insect was stabilized with forceps. While stabilizing the insect, an incision was made down the abdomen on the ventral side of the body using a sterile razor. The gut contents, which are brittle when frozen, were carefully picked out using fresh sterile forceps and placed in a sterile, pre-weighed 2 mL tube. Three guts were collected per tube.

### Culturing and identification of bacteria

An aliquot of 250 μl PBS/turgitol was added to each tube containing the dissected guts. Each tube was disrupted in a FastPrep FP120 instrument (Qbiogene) for 45 s, five times. 50 μl aliquots of neat suspension, 1:200 dilution and 1:4000 dilution were plated on LB media (LMM0202, Formedium), Reinforced Clostridia Agar (27,546, Sigma Chemicals), and MacConkey media (212,123, Difco). The plates were incubated for 24–48 h at 30 °C in aerobic conditions on LB and MacConkey media and in anaerobic conditions on Reinforced Clostridia Agar.

Each isolate was identified using 16S colony PCR with alkaline PEG reagent using 63f and 1389r primers and Taq DNA polymerase (28,104, Quiagen) [27]. DNA was sequenced using a BigDye v3.1 kit (Applied Bioscience) in a 10 μL reaction volume. The PCR products were sent to The Genome Analysis Centre (Earlham Institute, Norwich, UK) for processing. The sequences were trimmed to remove poorly recognized bases and run through the blastn algorithm (http://blast.ncbi.nlm.nih.gov/Blast.cgi) against the “Nucleotide collection (nr/nt)” database. Bacteria were identified if the sequence was ≥97% similar to 16S RNA gene in the database and had an e-value close or equal to 0.

### Identification of tetracycline-resistance elements

The gut samples with large gDNA fragments were subjected to diagnostic PCRs to establish if tetracycline genes were present. Ex Taq DNA polymerase (Clontech-Takara) was used in a 25 μL reaction volume, containing 10 ng template DNA. All reactions were carried out in a Mastercycler nexus X2 (Eppendorf). The initial denaturation was carried out at 94 °C for 10 min, followed by 35 cycles of denaturation (94 °C for 30 s), annealing (variable temperature for 1 min), and extension (72 °C for 2 min). The final extension was carried out at 72 °C for 10 min. The PCR products were separated by electrophoresis on 1% agarose Tris-acetate-EDTA gels.

### Metagenomic analyses

The genomic DNA (gDNA) was isolated from the lysed larval gut samples using FastDNA SPIN Kit for Soil (MP Biomedicals) according to the manufacturer’s instructions. The samples were separated by electrophoresis on 1% agarose TAE gels to confirm the presence of large gDNA fragments.

The gDNA isolated from the larval guts was sequenced at ChunLab (Seoul, South Korea) using a MiSeq Nano platform. The sequenced region was the V3-V4 region of the 16S gene. Taxonomic classification was assigned to each operational taxonomic unit at the species level using the ChunLab’s EzTaxon-e database and blastn algorithm [28]. Chimeric sequences were filtered out using the UCHIME program [29]. The sequencing results were analysed and visualised with CLcommunity software supplied by ChunLab.

## Additional files

Additional file 1: **Figure S1.** *Galleria mellonella* larvae feeding on artificial food in a Petri dish. (PDF 172 kb)

Additional file 2: **Table S1.** Overview of tet-resistance genes surveyed in the guts of *G. mellonella* larvae. (PDF 21 kb)

Additional file 3: **Figure S2.** Species richness in the guts of *G. mellonella* larvae feeding on artificial food with and without antibiotics. (PDF 18 kb)

Additional file 4: **Figure S3.** The composition of microbial communities identified in the guts of *G. mellonella* larvae. (PDF 43 kb)

## References

[1] Walsh C (2003). Antibiotics. Actions, origins, resistance.

[2] Martinez JL, Fajardo A, Garmendia L, Hernandez A, Linares JF, Martinez-Solano L, Sanchez MB (2009). A global view of antibiotic resistance. FEMS Microbiol Rev.

[3] D'Costa VM, King CE, Kalan L, Morar M, Sung WW, Schwarz C, Froese D, Zazula G, Calmels F, Debruyne R (2011). Antibiotic resistance is ancient. Nature.

[4] Segawa T, Takeuchi N, Rivera A, Yamada A, Yoshimura Y, Barcaza G, Shinbori K, Motoyama H, Kohshima S, Ushida K (2013). Distribution of antibiotic resistance genes in glacier environments. Environ Microbiol Rep.

[5] Allen HK, Cloud-Hansen KA, Wolinski JM, Guan C, Greene S, Lu S, Boeyink M, Broderick NA, Raffa KF, Handelsman J (2009). Resident microbiota of the gypsy moth Midgut harbors antibiotic resistance determinants. DNA Cell Biol.

[6] Tian B, Fadhil NH, Powell JE, Kwong WK, Moran NA. Long-term exposure to antibiotics has caused accumulation of resistance determinants in the gut microbiota of honeybees. mBio. 2012;3(6). 10.1128/mBio.00377-12 PMC3487773 23111871

[7] McKee BA, Goodman RD, Saywell C, Hepworth G (2003). Oxytetracycline hydrochloride activity in honey bee larvae (Apis mellifera) following medication with various doses. Apidologie.

[8] Waite R, Jackson S, Thompson H (2003). Preliminary investigations into possible resistance to oxytetracycline in Melissococcus plutonius, a pathogen of honeybee larvae. Lett Appl Microbiol.

[9] Peleg AY, Jara S, Monga D, Eliopoulos GM, Moellering RC, Mylonakis E (2009). Galleria mellonella as a model system to study Acinetobacter baumannii pathogenesis and therapeutics. Antimicrob Agents Chemother.

[10] Ignasiak K, Maxwell A (2017). Galleria mellonella (greater wax moth) larvae as a model for antibiotic susceptibility testing and acute toxicity trials. BMC Res Notes.

[11] Waterhouse DF (1959). Axenic culture of wax moths for digestion studies. Ann N Y Acad Sci.

[12] Stephens JM (1962). A strain of Streptococcus Faecalis Andrewes and Horder producing mortality in larvae of galleria Mellonella (Linnaeus). J Insect Pathol.

[13] Johnston PR, Rolff J (2015). Host and Symbiont jointly control gut microbiota during complete metamorphosis. PLoS Pathog.

[14] Dudziak B (1975). Studies on the role of microorganisms in alimentation of galleria mellonella larvae. Annales Universitatis Mariae Curie-Sklodowska, Section C.

[15] Lebreton F, van Schaik W, Sanguinetti M, Posteraro B, Torelli R, Le Bras F, Verneuil N, Zhang X, Giard JC, Dhalluin A (2012). AsrR is an oxidative stress sensing regulator modulating enterococcus faecium opportunistic traits, antimicrobial resistance, and pathogenicity. PLoS Pathog.

[16] Krams I, Kecko S, Inashkina I, Trakimas G, Krams R, Elferts D, Vrublevska J, Joers P, Rantala MJ, Luoto S (2017). Food quality affects the expression of antimicrobial peptide genes upon simulated parasite attack in the larvae of greater wax moth. Entomol Exp Appl.

[17] Krams IA, Kecko S, Joers P, Trakimas G, Elferts D, Krams R, Luoto S, Rantala MJ, Inashkina I, Gudra D (2017). Microbiome symbionts and diet diversity incur costs on the immune system of insect larvae. J Exp Biol.

[18] Kulkarni N, Kushwaha DK, Mishra VK, Paunikar S (2012). Effect of economical modification in artificial diet of greater wax moth galleria mellonella (Lepidoptera: pyralidae). Indian J Entomol.

[19] Buyukguzel E, Kalender Y (2008). Galleria mellonella (L.) survivorship, development and protein content in response to dietary antibiotics. J Entomol Sci.

[20] Cho SH, Lim YS, Kang YH (2012). Comparison of antimicrobial resistance in Escherichia coli strains isolated from healthy poultry and swine farm workers using antibiotics in Korea. Osong Public Health Res Perspect.

[21] Marshall BM, Levy SB (2011). Food animals and antimicrobials: impacts on human health. Clin Microbiol Rev.

[22] Jarosz J (1979). Gut flora of galleria mellonella suppressing ingested bacteria. J Invertebr Pathol.

[23] Jarosz J (1983). Alterations in Cathodal protein-fractions in immune wax moth Pupal blood. Cytobios.

[24] Donhofer A, Franckenberg S, Wickles S, Berninghausen O, Beckmann R, Wilson DN (2012). Structural basis for TetM-mediated tetracycline resistance. Proc Natl Acad Sci U S A.

[25] Chopra I, Roberts M (2001). Tetracycline antibiotics: mode of action, applications, molecular biology, and epidemiology of bacterial resistance. Microbiol Mol Biol Rev.

[26] Roberts MC. Tetracycline Resistance Due to Ribosomal Protection Proteins. In: White DG, Alekshun MN, Mcdermott PF, editors. *Frontiers in Antimicrobial Resistance: A Tribute to Stuart B Levy*. Washington: American Society for Microbiology; 2005.

[27] Chomczynski P, Rymaszewski M (2006). Alkaline polyethylene glycol-based method for direct PCR from bacteria, eukaryotic tissue samples, and whole blood. Biotechniques.

[28] Kim OS, Cho YJ, Lee K, Yoon SH, Kim M, Na H, Park SC, Jeon YS, Lee JH, Yi H (2012). Introducing EzTaxon-e: a prokaryotic 16S rRNA gene sequence database with phylotypes that represent uncultured species. Int J Syst Evol Microbiol.

[29] Edgar RC, Haas BJ, Clemente JC, Quince C, Knight R (2011). UCHIME improves sensitivity and speed of chimera detection. Bioinformatics.

