# Supplementary figures and images for: Oxytetracycline reduces the diversity of tetracycline-resistance genes in the Galleria mellonella gut microbiome
Source: BMC Microbiol. 2018 Dec 29;18:228. doi: 10.1186/s12866-018-1377-3 (PMC6310997; doi:10.1186/s12866-018-1377-3)

**Figure S1.** *Galleria mellonella* larvae feeding on artificial food in a Petri dish.

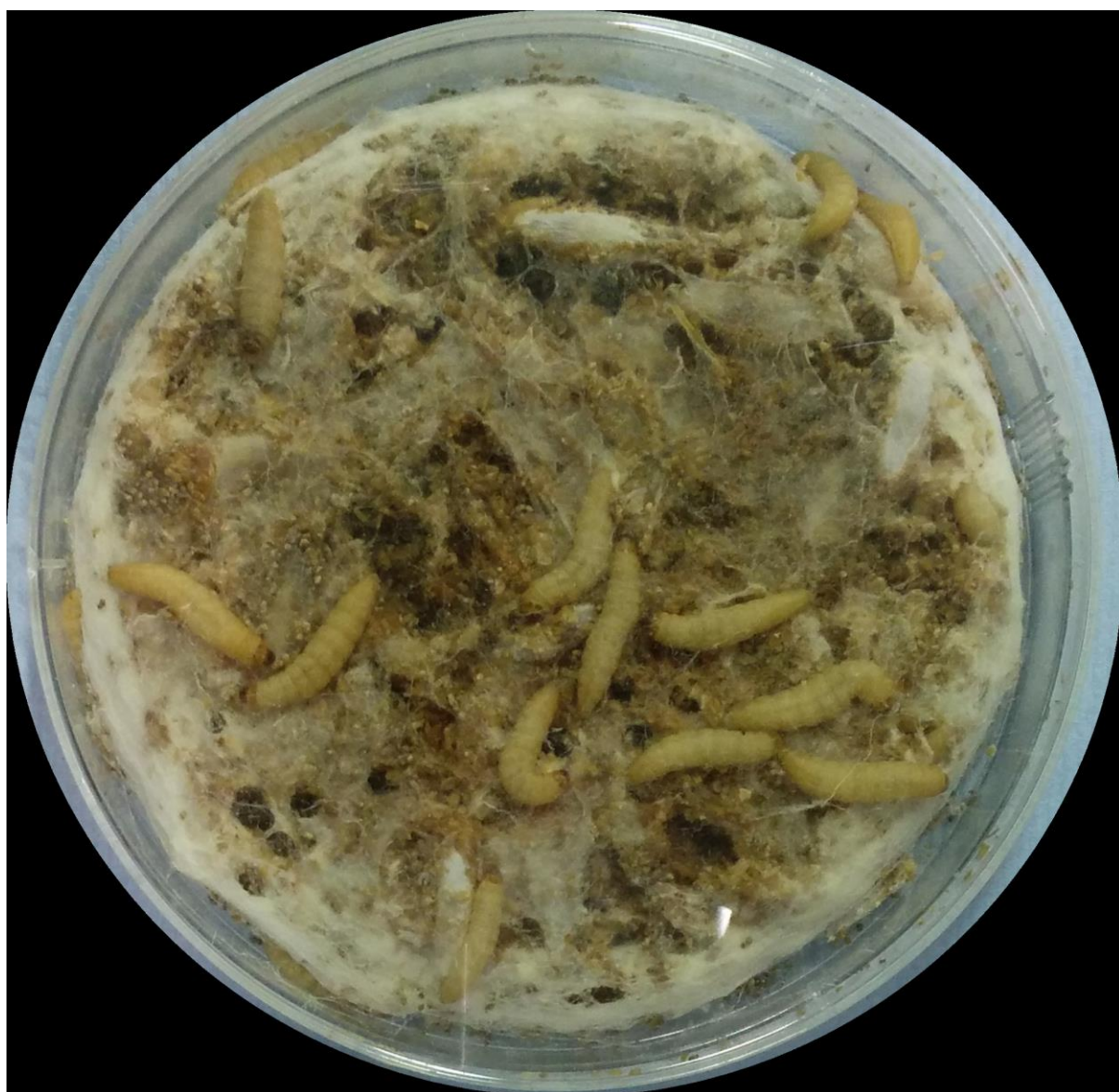

Supplement: Supplementary file 1 — Figure S1. Galleria mellonella larvae feeding on artificial food in a Petri dish. (PDF 172 kb) [file 12866_2018_1377_MOESM1_ESM.pdf]
