# Supplementary material for: Oxytetracycline reduces the diversity of tetracycline-resistance genes in the Galleria mellonella gut microbiome
Source: BMC Microbiol. 2018 Dec 29;18:228. doi: 10.1186/s12866-018-1377-3 (PMC6310997; doi:10.1186/s12866-018-1377-3)
Supplement: Supplementary file 2 — Table S1. Overview of tet-resistance genes surveyed in the guts of G. mellonella larvae. (PDF 21 kb) [file 12866_2018_1377_MOESM2_ESM.pdf]

Table S1. Overview of tet-resistance genes surveyed in the guts of *G. mellonella* larvae.

gene name	gene product	reference
tetA	efflux pump	Fan *et al*., 2007 [1]
tetB	efflux pump	Fan *et al*., 2007 [1]
tetC	efflux pump	Fan *et al*., 2007 [1]
tetD	efflux pump	Fan *et al*., 2007 [1]
tetE	efflux pump	Fan *et al*., 2007 [1]
tetG	efflux pump	Fan *et al*., 2007 [1]
tetH	efflux pump	Fan *et al*., 2007 [1]
tetJ	efflux pump	Aminov *et al*., 2002, 2004 [2, 3]
tetK	efflux pump	Chopra and Roberts, 2001 [4]
tetL	efflux pump	Tian *et al*., 2012 [5]
tetY	efflux pump	Aminov *et al*., 2002, 2004 [2, 3]
tetZ	efflux pump	Aminov *et al*., 2002, 2004 [2, 3]
tet30	efflux pump	Aminov *et al*., 2002, 2004 [2, 3]
tetBP	ribosomal protection protein	Aminov *et al*., 2002, 2004 [2, 3]
tetM	ribosomal protection protein	Aminov *et al*., 2002, 2004 [2, 3]
tetO	ribosomal protection protein	Aminov *et al*., 2002, 2004 [2, 3]
tetQ	ribosomal protection protein	Aminov *et al*., 2002, 2004 [2, 3]
tetS	ribosomal protection protein	Aminov *et al*., 2002, 2004 [2, 3]
tetT	ribosomal protection protein	Aminov *et al*., 2002, 2004 [2, 3]
tetW	ribosomal protection protein	Aminov *et al*., 2002, 2004 [2, 3]
tet32	ribosomal protection protein	Aminov *et al*., 2002, 2004 [2, 3]
tet34	efflux pump	Szczepanowski *et al*., 2009 [6]
tetX	tetracycline destructase	Bartha *et al*., 2011 [7]
tetX2	tetracycline destructase	Bartha *et al*., 2011 [7]

References:

1. Fan W, Hamilton T, Webster-Sesay S, Nikolich MP, Lindler LE: **Multiplex real-time SYBR Green I PCR assay for detection of tetracycline efflux genes of Gram-negative bacteria**. *Mol Cell Probes* 2007, **21**(4):245-256.
2. Aminov RI, Chee-Sanford JC, Garrigues N, Mehboob A, Mackie RI: **Detection of tetracycline resistance genes by PCR methods**. *Methods Mol Biol* 2004, **268**:3-13.
3. Aminov RI, Chee-Sanford JC, Garrigues N, Teferedegne B, Krapac IJ, White BA, Mackie RI: **Development, validation, and application of PCR primers for detection of tetracycline efflux genes of gram-negative bacteria**. *Appl Environ Microbiol* 2002, **68**(4):1786-1793.
4. Chopra I, Roberts M: **Tetracycline antibiotics: mode of action, applications, molecular biology, and epidemiology of bacterial resistance**. *Microbiol Mol Biol Rev* 2001, **65**(2):232-260.
5. Tian B, Fadhil NH, Powell JE, Kwong WK, Moran NA: **Long-term exposure to antibiotics has caused accumulation of resistance determinants in the gut microbiota of honeybees**. *mBio* 2012, **3**(6).
6. Szczepanowski R, Linke B, Krahn I, Gartemann KH, Gutzkow T, Eichler W, Puhler A, Schluter A: **Detection of 140 clinically relevant antibiotic-resistance genes in the plasmid metagenome of wastewater treatment plant bacteria showing reduced susceptibility to selected antibiotics**. *Microbiology* 2009, **155**(Pt 7):2306-2319.
7. Bartha NA, Soki J, Urban E, Nagy E: **Investigation of the prevalence of tetQ, tetX and tetX1 genes in Bacteroides strains with elevated tigecycline minimum inhibitory concentrations**. *International journal of antimicrobial agents* 2011, **38**(6):522-525.
