# Supplementary material for: Oxytetracycline reduces the diversity of tetracycline-resistance genes in the Galleria mellonella gut microbiome
Source: BMC Microbiol. 2018 Dec 29;18:228. doi: 10.1186/s12866-018-1377-3 (PMC6310997; doi:10.1186/s12866-018-1377-3)

Figure S2. Species richness in the guts of *G. mellonella* larvae feeding on artificial food with (blue line) and without (red line) antibiotics. The rarefaction curve shows the rate of increase in the number of species that are discovered as more reads are being sequenced. More different bacterial strains were identified in the guts of *G. mellonella* feeding on artificial food with oxytetracycline than in the guts of larvae not exposed to the antibiotic.

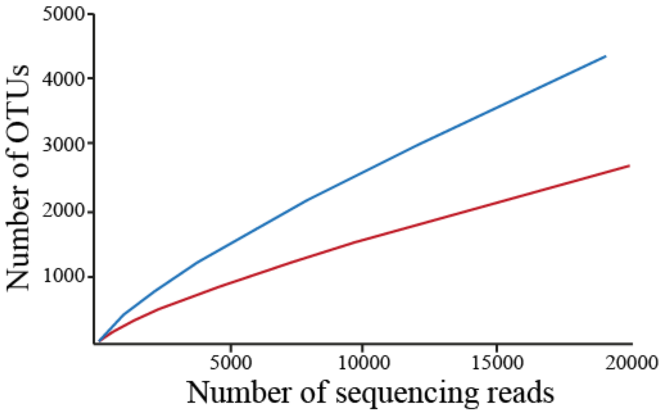

Supplement: Supplementary file 3 — Figure S2. Species richness in the guts of G. mellonella larvae feeding on artificial food with and without antibiotics. (PDF 18 kb) [file 12866_2018_1377_MOESM3_ESM.pdf]
