# Supplementary material for: Oxytetracycline reduces the diversity of tetracycline-resistance genes in the Galleria mellonella gut microbiome
Source: BMC Microbiol. 2018 Dec 29;18:228. doi: 10.1186/s12866-018-1377-3 (PMC6310997; doi:10.1186/s12866-018-1377-3)

Figure S3. The composition of microbial communities identified in the guts of *G. mellonella* larvae. Each chart represents a sequenced sample, obtained by pooling three larval guts. a) Microbial community from the guts of larvae feeding on artificial food with no antibiotic. Firmicutes is the only phylum present in the gut. The gut community is dominated by *Enterococcus mundtii* and closely-related Enterococcaceae. b) Five different bacterial phyla are present in the guts of larvae feeding on artificial food containing oxytetracycline at 400 mg/100 g food. Genus Enterococcaceae is not the only genus present in the phylum Firmicutes. Only phylogenetic groups with abundance over 1% have been shown.

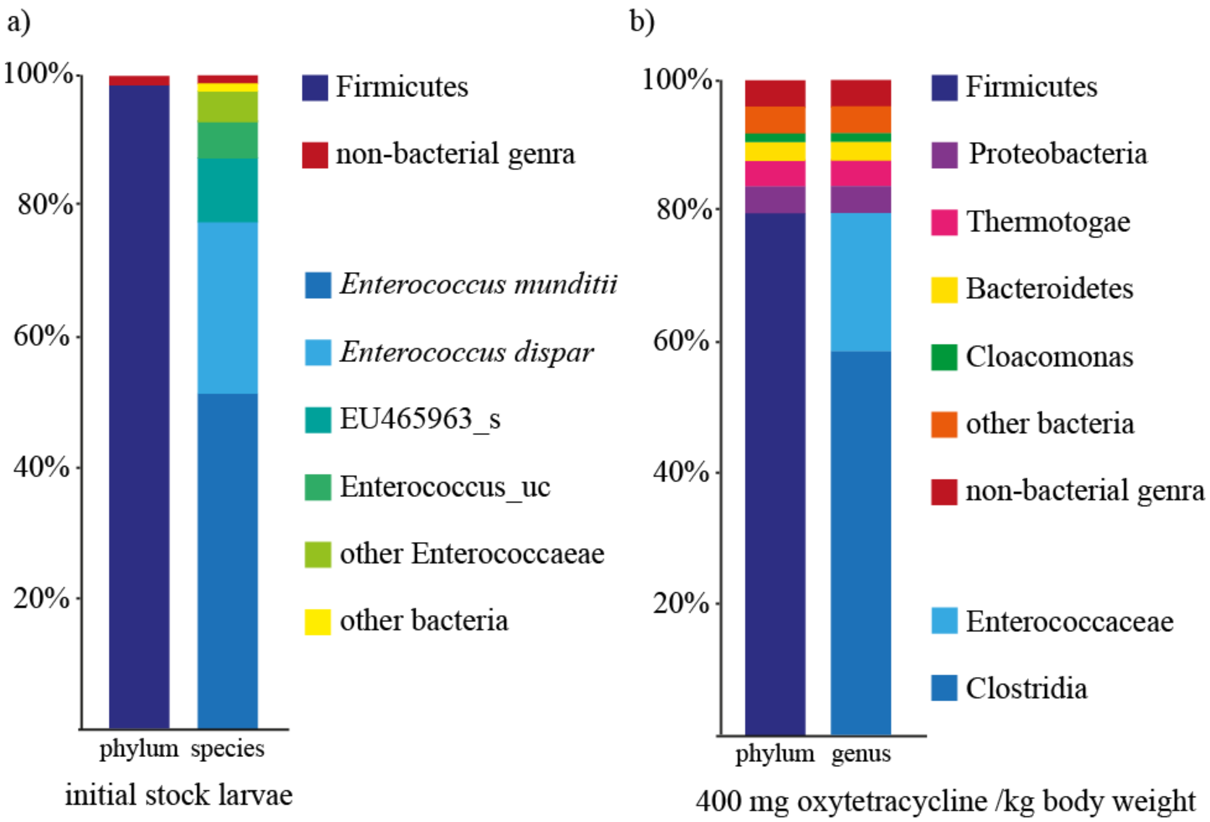

Supplement: Supplementary file 4 — Figure S3. The composition of microbial communities identified in the guts of G. mellonella larvae. (PDF 43 kb) [file 12866_2018_1377_MOESM4_ESM.pdf]
